# Effects of isoflavone-supplemented soy yogurt on lipid parameters and atherosclerosis development in hypercholesterolemic rabbits: a randomized double-blind study

**DOI:** 10.1186/1476-511X-8-40

**Published:** 2009-10-08

**Authors:** Daniela CU Cavallini, Dulcinéia SP Abdalla, Regina C Vendramini, Raquel Bedani, Laura Q Bomdespacho, Nadiége D Pauly-Silveira, Graciela F de Valdez, Elizeu A Rossi

**Affiliations:** 1 Department of Food & Nutrition, Faculty of Pharmaceutical Sciences, São Paulo State University, Araraquara, SP, Brazil; 2 Department of Clinical and Toxicological Analyses, Faculty of Pharmaceutical Sciences, University of Sao Paulo, Sao Paulo, Brazil; 3 Department of Clinical Analysis, Faculty of Pharmaceutical Sciences, São Paulo State University, Araraquara, SP, Brazil; 4 Reference Center for Lactobacilos, CERELA, SM Tucumán, Argentina

## Abstract

**Background:**

There is increasing interest in natural treatments to control dyslipidemia and reduce the risk of cardiovascular disease. Previous studies have demonstrated the beneficial effects of soy yogurt fermented with *Enterococcus faecium *CRL 183 and of dietary isoflavones on the lipid profile. The purpose of the present study was to investigate the effects of isoflavone-supplemented soy yogurt, fermented with *E. faecium *CRL183, on lipid parameters and atherosclerosis development in rabbits with induced hypercholesterolemia.

**Methods:**

Forty-eight rabbits were randomly assigned to eight groups fed on the following diets for 60 days: C - control; IY - isoflavone-supplemented soy yogurt; H - hypercholesterolemic (1.0% cholesterol wt/wt diet); HY - hypercholesterolemic plus soy yogurt; HIY - hypercholesterolemic plus isoflavone-supplemented soy yogurt; HP - hypercholesterolemic plus placebo; HI - hypercholesterolemic plus isoflavone and HE - hypercholesterolemic plus pure culture of *E. faecium *CRL 183. Serum lipids and autoantibodies against oxLDL (oxLDL Ab) were analyzed on days 0, 30 and 60 of the treatment and the atherosclerotic lesions were quantified at the end of the experiment.

**Results:**

Soy yogurt, soy yogurt supplemented with isoflavones and placebo promoted significant reductions in total cholesterol level (38.1%, 27.0% and 26.6%, respectively). Significant increases in serum HDL-C concentration relative to group H were detected in animals that ingested soy yogurt, with or without the isoflavone supplement (55.2%), *E. faecium *culture (43.3%) or placebo (35.8%). Intake of soy yogurt and soy yogurt supplemented with isoflavones prevented the rise of oxLDL Ab during the study period. The extent of atherosclerosis in the thoracic and abdominal aortas was reduced in the HIY, HY and HP groups. However, when the whole aorta was analyzed, animals treated with soy yogurt supplemented with isoflavones exhibited the greatest reduction (51.4%, P < 0.05) in atherosclerotic lesion area, compared to group H.

**Conclusion:**

Soy yogurt could be consumed as an alternative means of reducing the risk of cardiovascular disease by improving the lipid profile and inhibiting oxLDL Ab formation. Our findings also suggest that isoflavone supplementation may enhance the antiatherosclerotic effect of soy yogurt.

## Background

Atherosclerosis is a vascular chronic inflammation in the arterial wall that can lead to clinical manifestations including myocardial infarction, peripheral arterial disease and stroke [1]. Although multiple risk factors have been shown to play a significant role in the pathogenesis of atherosclerosis and cardiovascular disease (CVD), dyslipidemia remains a major determining factor for these pathologies. Epidemiological studies have associated high levels of total cholesterol and LDL and reduced concentrations of HDL with the risk of experiencing future cardiovascular events. Studies have shown that for every 1.0% reduction in blood cholesterol there is a corresponding 2.5-3.0% reduction in the incidence of heart disease [2,3]. Additionally, oxidative modification of LDL plays an important role in the development of atherosclerosis. Oxidized LDLs are readily taken up by the scavenger receptors in macrophages, leading to the formation of foam cells, modulation of the expression of growth factors, adhesion molecules and cytokines and stimulation of circulating monocytes. Oxidation of LDL also leads to the formation of autoantibodies against oxidized LDL (oxLDL), whose proatherogenic or antiatherogenic effects have been extensively studied [4-8]. Therefore, the control of cholesterol level and of oxidative modification of lipoproteins could lower the occurrence of CVD.

There is a growing interest in the dietary management of dyslipidemias, driven by the high cost of drug therapy and the side effects of such treatment. The dietary components that may reduce blood cholesterol and protect against CVD include soluble fibers, soy proteins, isoflavones and probiotic microorganisms [9-12].

Several *in vitro *and *in vivo *studies have shown that the cardioprotective effects of soy isoflavones include an improvement in serum lipid profiles [10,13] and in vascular reactivity [14], protection of LDL against oxidation [15], modulation of cytokines and inhibition of platelet aggregation [14].

In an *in vitro *experiment, we demonstrated that *E. faecium *CRL183 reduces cholesterol by 53.85% [16]. We have also reported that a soy yogurt fermented with *E. faecium *CRL 183 and *Lactobacillus helveticus *ssp *jugurti *416 was able to improve the lipid parameters in animal and human tests [17-19]. However, during the processing of soy to produce the soy yogurt, the total isoflavone content is reduced by 92% compared to the original level in the whole soybean [20]. The supplementation of the soy yogurt with isoflavones, to bring the content to approximately that in the whole bean, presumably reinforces the beneficial effects of the fermented product.

To test this hypothesis, in the present study we have investigated the effects of a soy yogurt, fermented with *E. faecium *CRL 183 and supplemented with isoflavones, on the lipid profile and atherosclerosis development in rabbits with diet-induced hypercholesterolemia.

## Methods

### Animals and experimental protocol

All studies were performed with the approval of the Research Ethics Committee of the School of Pharmaceutical Sciences (UNESP at Araraquara, SP, Brazil).

A total of 48 New Zealand White male rabbits (from the Central Animal Facility of Sao Paulo State University, Botucatu, SP, Brazil), 8-9 weeks old and weighing 2.5-3.0 kg, were housed individually in temperature-controlled rooms (22°C) with a light-dark cycle of 12:12 h.

Rabbits were randomly divided into the following eight dietary groups (n = 6): control (C), hypercholesterolemic (H), isoflavone-supplemented soy yogurt (IY), hypercholesterolemic plus soy yogurt (HY), hypercholesterolemic plus isoflavone-supplemented soy yogurt (HIY), hypercholesterolemic plus placebo (HP), hypercholesterolemic plus isoflavone (HI) and hypercholesterolemic plus *E. faecium *CRL 183 (HE).

The control (C) and isoflavone-supplemented soy yogurt (IY) groups were fed on commercial rabbit food (Nutri Coelhos Especial, Purina, Brazil), with the following nutritional make-up (per 100 g): 23 g protein, 4 g fats, 49 g carbohydrates, 5 g fiber and 10 g minerals. The other groups (H, HY, HIY, HP, HI and HE) were fed on the same rabbit diet, to which cholesterol (Sigma Chemical Co C 8503) had been added to induce hypercholesterolemia. The level of cholesterol added to the diet was adjusted during the experimental period (from 1.0% to 0.7% after 30 days) to maintain the health of the animal. To prepare the supplemented diet, cholesterol dissolved in ether (ethyl ether stabilized with BHT, Carlo Erba, Italy), was spraying onto the chow, under a hood, where it remained for 12 h to allow the solvent to evaporate completely. The chow was stored at -10°C for no more than 2 weeks before use. The rabbits received restricted amounts (125 g/d) of each diet, because the extent of atherosclerosis depends on total cholesterol intake [9]. The animals had free access to water during the experimental period. Groups HY, HIY, HP, HE and HI were given, by gavage once a day, soy yogurt (2.8 mL/kg body weight), isoflavone-supplemented soy yogurt (2.8 mL/kg - 2.1 mg of total isoflavone/kg body weight), placebo (2.8 mL/kg body weight), *E. faecium *suspension (10^8 ^- 10^9 ^CFU) and isoflavone (2.1 mg/kg body weight), respectively.

The soy yogurt was processed at UNISOJA (Development and Production Unit for Soybean Derivatives) in the Food and Nutrition Department of the School of Pharmaceutical Sciences, UNESP at Araraquara (SP, Brazil), following the method described by Rossi *et al*. [17]. The conventional bacterial inoculum was replaced by 3% (v/v) of a 1:1 mixture of exponential phase cultures of *Enterococcus faecium *CRL 183 (probiotic microrganism) and *Lactobacillus helveticus *ssp *jugurti *416 (fermentation adjuvant). Chemical analysis of the product showed that 100 g provided: 86.4 kcal, 3.39 g protein, 2.74 g lipids, 12.05 g carbohydrates, 0.47 g ash, 81.32 g water and 5.24 mg total isoflavones (0.98 mg daidzin, 0.37 mg glycitin, 3.72 mg genistin, 0.04 mg daidzein, 0.04 mg glycitein and 0.09 mg genistein). Isoflavin^® ^(Galena, Brazil) was added to the soy yogurt before the fermentation, at 75 mg (total isoflavone) per 100 g, to yield the isoflavone-supplemented soy yogurt. Isoflavin^® ^contains at least 42.3% total isoflavones, which consist of 4.7% genistin, 11.3% genistein, 5.5% daidzin, 17.8% daidzein, 2.0% glycitin and 1.0% glycitein. Placebo was prepared by chemical acidification (with lactic acid) of soy yogurt basic mixture (without bacterial culture or isoflavone). To prepare the pure culture of probiotic microorganism, *E. faecium *CRL 183 was inoculated into Tryptic Soy Broth (Acumedia) and incubated at 37°C for 16 hours. The cells were centrifuged at 3000 rpm for 5 minutes and the supernatant discarded. The cells were resuspended in sterile peptone water and the suspension was stored under refrigeration until administered to the animals. Soy yogurt, isoflavone-supplemented soy yogurt and pure culture of probiotic microorganism exhibited counts between 10^8 ^- 10^9 ^CFU/mL. Isoflavone mixture was diluted in sterile water immediately before use.

All animals were fed the experimental diets for 60 days and were weighed 3 times during the study (0, 30 and 60 days). Food intake was measured daily.

### Blood Sampling

Blood was drawn from the marginal ear vein, after a 14 to 16-hour fast, on days 0, 30 and 60 of treatment. The samples were centrifuged (3500 × g for 10 min) and the serum was taken for lipid profile determination. For the oxLDL Ab determination, plasma was first separated from the blood (collected into tubes containing 1.0 g/L EDTA) by centrifugation (3500 × g for 10 min at 4°C), then 1.0 mmol/L phenylmethylsulfonyl fluoride (Sigma Chemical), 2.0 mmol/L benzamidine (Sigma Chemical), 2.0 mg/L aprotinin (Sigma Chemical) and 20.0 mmol/L BHT (Sigma Chemical) were added to the samples of plasma.

### Analysis of Serum lipids

The serum levels of TC, HDL-C and triglycerides were assayed in each rabbit, with the aid of specific enzyme kits. Total cholesterol was measured by the cholesterol fast color method [21]. HDL cholesterol was estimated by first selectively precipitating lipoproteins [22] and then applying the TC method to the supernatant. Triglycerides were measured by the triglyceride fast color method [23]. Non-HDL cholesterol was calculated by subtracting HDL-C from TC and was composed of the LDL+IDL+VLDL cholesterol fractions [24,25].

### Detection of autoantibodies against ox LDL (oxLDL Ab)

The autoantibodies against ox LDL (oxLDL Ab) were detected in plasma as described by Damasceno et al [5] with modifications. Aliquots of native LDL and electronegative LDL were diluted in carbonate-bicarbonate buffer (0.1 M, pH 9.4) to 1.0 μg of protein/well and used to coat 96-well ELISA microplates (Costar, Cambridge, MA, USA) overnight at 4°C. Next, microplates were blocked with 5% fat free milk in phosphate-buffered saline (PBS) pH 7.4, previously inactivated by heating (100°C), and incubated at 37°C for 30 min. After washing the plates 3 times with PBS-Tween (0.05%), plasma samples (50 μL/well) previously diluted in PBS-1% fat free milk (1:10) were added and plates were incubated for 2 h at room temperature. After washing as previously described, peroxidase-conjugated affinity goat polyclonal anti-rabbit IgG (Rockland, diluted 1:2000 in PBS-1% fat free milk, 50 μL/well) was added and plates were incubated for 1.5 h at room temperature. After incubation, plates were washed 3 times with PBS-Tween (0.05%) and peroxidase substrate (0.53 mg ABTS/mL in citrate phosphate buffer pH 4.5 plus H_2_O_2_) was added. The microplates were incubated for 30 min at room temperature and absorbance was monitored at 405 nm with a SpectraCount^® ^microplate photometer (Packard BioScience Company). The intra-assay and inter-assay variations for this ELISA were 6% and 10%, respectively. Results were shown as the mean of absorbance values of triplicates with variation ≤ 10%. The data were presented as mg/L.

### Morphometric examination of lesions

At the end of the experiments, the rabbits were heparinized and euthanized by an overdose of sodium phenobarbital (Cristália, SP, Brazil). After euthanasia, the liver, kidneys and heart were removed and weighed. The aorta was removed from the aortic valve to the iliac bifurcation to analyze the atherosclerotic lesions. The aorta was divided into two segments, comprising: 1) aortic arch; 2) thoracic aorta and abdominal aorta. The material was fixed in 10% neutral buffered formaldehyde (overnight, 37°C), rinsed with saline solution and stained with Sudan IV to visualize areas of atherosclerotic plaque [26]. The stained aorta was photographed with a digital camera (Sony) and the sudanophilic lesions were identified and quantified. The surface area of the atherosclerotic lesions was measured with an image analyzer system (Imagelab - USP, Brazil) and expressed as the percentage of the total surface area of the aortic intima covered by lesion.

### Statistical analysis

Quantitative results were reported as mean ± SEM. The data were tested by analysis of variance (ANOVA) and the means were compared across groups by the Tukey test, significance being declared when P ≤ 0.05. All analyses were carried out with the BIOSTAT statistical package.

## Results

### Food intake, weight gain and organ weight

Food intake was reduced in the animals of groups HIY and HI, without differing significantly from placebo (HP) or *E. faecium *(HE) - treated animals. Despite differences in food intake, the weight gain showed no difference between groups (P < 0.05). The rabbits fed only a hypercholesterolemic diet (group H) showed the highest weights for liver, heart and kidneys (Table 1).

**Table 1:** Effects of different treatments on food intake, body weight and organ weight in the rabbits

Groups*	**Food Intake (g)**	**Weight gain (g)**	**Liver (g)**	**Heart (g)**	**Kidney (g)**
**C**	125.00 ± 0.00^a^	903.05 ± 124.86^a^	83.17 ± 3.92^d^	5.78 ± 0.20^b^	6.59 ± 0.52^d^
**IY**	125.00 ± 0.00^a^	760.08 ± 44.16^a^	97.22 ± 8.39^cd^	6.06 ± 0.24^ab^	7.47 ± 0.10^c^
**H**	124.93 ± 0.50^a^	713.40 ± 39.75^a^	165.90 ± 3.41^a^	7.06 ± 0.56^a^	10.02 ± 0.72^a^
**HIY**	113.76 ± 24.90^b^	738.68 ± 144.36^a^	111.44 ± 7.21^c^	6.69 ± 0.48^ab^	7.67 ± 0.33^cd^
**HY**	122.57 ± 8.19^a^	680.50 ± 64.23^a^	146.08 ± 2.51^ab^	6.55 ± 0.16^ab^	8.39 ± 0.28^b^
**HP**	119.46 ± 10.21^ab^	824.50 ± 95.87^a^	134.61 ± 3.12^bc^	6.84 ± 0.06^ab^	9.30 ± 0.14^a^
**HE**	118.17 ± 9.92^ab^	810.57 ± 95.22^a^	110.12 ± 7.53^c^	6.24 ± 0.39^ab^	7.21 ± 0.46^cd^
**HI**	110.14 ± 17.52^b^	851.88 ± 67.04^a^	98.22 ± 6.07^cd^	5.73 ± 0.22^b^	7.36 ± 0.74^cd^

Values represent mean ± SEM (n = 6).

Statistical comparison of groups: means with identical letters in the same column do not differ significantly (P ≤ 0.05).

* C = control; IY = isoflavone-supplemented soy yogurt; H = hypercholesterolemic; HIY = hypercholesterolemic plus isoflavone-supplemented soy yogurt; HY = hypercholesterolemic plus soy yogurt; HP = hypercholesterolemic plus placebo; HE = hypercholesterolemic plus *E.faecium*; HI = hypercholesterolemic plus isoflavone.

### Effects on serum lipids

The effects of treatments on serum lipids are shown in Table 2. All animals had comparable serum levels of TC, HDL-C and n-HDL-C at the beginning of the experiment. After 30 days, group H showed the highest concentrations of TC and n-HDL-C, without differing from groups HIY, HP and HE. The rabbits that received soy yogurt exhibited the greatest reduction (P < 0.05) in TC (32.8%) and n-HDL-C (33.2%), relative to group H. Isoflavones lowered the TC and n-HDL-C levels by 19.5% and 19.8%, respectively. At the end of the experiment - 30 days after the reduction of the cholesterol added to the chow (from 1% to 0.7%) - groups H, HE and HI showed the highest TC and n-HDL-C concentrations without differing among themselves. Soy yogurt, soy yogurt supplemented with isoflavones and placebo promoted significant reductions in TC level (38.1%, 27.0% and 26.6% respectively).

**Table 2:** Serum lipid levels in rabbits during the experiment.

**Time**	**C**	**IY**	**H**	**HIY**	**HY**	**HP**	**HE**	**HI**
**TC**								

**Initial**	49.0 ± 3.7^a^	42.0 ± 4.4^a^	42.0 ± 2.8^a^	44. 5 ± 5.4^a^	45.3 ± 5.1^a^	44.8 ± 5.8^a^	46.3 ± 2.7^a^	46.0 ± 1.9^a^
**T30**	63.3 ± 4.3^d^	59.3 ± 6.6^d^	3650.0 ± 138.0^a^	3517.5 ± 159.9^a^	2452.5 ± 277.5^c^	3380.0 ± 189.9^ab^	3413.5 ± 192.7^a^	2938.5 ± 154.5^b^
**T60**	53.3 ± 6.2^c^	43.5 ± 4.4^c^	2556.5 ± 120.3^a^	1867.5 ± 251.7^b^	1583.8 ± 86.3^b^	1876.3 ± 233.6^b^	2605.0 ± 166.2^a^	2327.5 ± 71.5^a^

**HDL-C**								

**Initial**	31.5 ± 2.3^a^	29.8 ± 2.2^a^	28.5 ± 2.5^a^	31.8 ± 1.1^a^	32.0 ± 2.6^a^	31.5 ± 3.6^a^	30.3 ± 1.5^a^	29.8 ± 1.9^a^
**T30**	38.3 ± 2.1^a^	36.8 ± 3.8^ab^	25.5 ± 0.9^c^	37.3 ± 2.4^ab^	31.0 ± 2.2^bc^	32.0 ± 2.5^abc^	34.3 ± 2.7^ab^	30.8 ± 1.8^bc^
**T60**	26.8 ± 2.8^a^	27.0 ± 1.7^a^	16.8 ± 2.8^c^	26.0 ± 2.1^a^	26.0 ± 1.7^a^	22.8 ± 2.8^ab^	24.0 ± 1.9^a^	20.5 ± 2.1^bc^

**n-HDL-C**								

**Initial**	17.5 ± 2.6^a^	12.3 ± 2.8^a^	13.5 ± 2.9^a^	12.8 ± 2.3^a^	13.3 ± 3.0^a^	13.3 ± 2.4^a^	16.0 ± 2.0^a^	16.3 ± 1.92^a^
**T30**	25.0 ± 2.6^d^	22.5 ± 2.9^d^	3624.5 ± 138.0^a^	3480.3 ± 162.2^a^	2421.5 ± 278.5^c^	3348.0 ± 187.4^ab^	3379.3 ± 193.1^a^	2907.8 ± 154.5^b^
**T60**	26.5 ± 4.4^c^	16.5 ± 2.5^c^	2539.8 ± 120.2^a^	1841.5 ± 249.8^b^	1557.8 ± 86.6^b^	1853.5 ± 231.8^b^	2581.5 ± 166.2^a^	2307.0 ± 71.3^a^

**TGL**								

**Initial**	90.0 ± 5.3^ab^	68.5 ± 12.8^b^	66.0 ± 7.3^b^	65.8 ± 7.2^b^	61.5 ± 8.9^b^	59.3 ± 4.4^b^	99.5 ± 9.5^a^	99.3 ± 9.2^a^
**T30**	57.5 ± 2.9^e^	83.5 ± 4.2^de^	260.3 ± 9.4^a^	145.5 ± 27.5^c^	245.3 ± 17.6^ab^	262.0 ± 31.8^a^	122.8 ± 20.4^cd^	203.3 ± 3.6^b^
**T60**	53.0 ± 4.5^d^	61.0 ± 7.3^d^	245.5 ± 25.9^a^	70.5 ± 3.9^d^	235.0 ± 27.7^a^	192.0 ± 12.3^b^	115.8 ± 6.4^c^	215.0 ± 7.1^ab^

Values (in mg/dL) represent mean ± SEM (n = 6). Statistical comparison of groups: means with identical letters on the same line do not differ significantly (P ≤ 0.05).

C = control; IY = isoflavone supplemented soy yogurt; H = hypercholesterolemic; HIY = hypercholesterolemic plus isoflavone-supplemented soy yogurt; HY = hypercholesterolemic plus soy yogurt; HP = hypercholesterolemic plus placebo; HE = hypercholesterolemic plus *E.faecium*; HI = hypercholesterolemic plus isoflavone.

TC = Total Cholesterol; HDL-C = High Density Lipoprotein; n-HDL-C = non - High Density Lipoprotein; TGL = Triglycerides.

HDL-C concentration was lower (P < 0.05) in the animals of groups H, HY, HP and HI, after 30 days of treatment. On the other hand, rabbits in HIY, IY and HE showed levels of HDL-C significantly higher (46.1%, 44.1% and 34.3%, respectively) than group H and similar to the control. At the end of the study, a significant increase in HDL-C was detected in animals that received the soy yogurt, with or without isoflavone supplement (55.2%), probiotic microorganism (43.3%) or placebo (35.8%), relative to group H. After 60 days of study, groups HIY, HP and HE had lower triglyceride levels (P < 0.05) than group H.

### Autoantibodies against oxLDL (oxLDL Ab)

**Figure 1:**
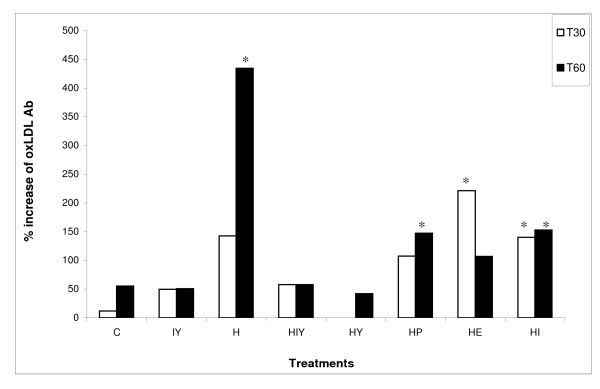
**Changes in autoantibodies against oxLDL (percentage) in plasma of rabbits, compared with the baseline levels**. Values are means, n = 6. * Significantly different from day 0 (P < 0.05). C = control; IY = isoflavone supplemented soy yogurt; H = hypercholesterolemic; HIY = hypercholesterolemic plus isoflavone-supplemented soy yogurt; HY = hypercholesterolemic plus soy yogurt; HP = hypercholesterolemic plus placebo; HE = hypercholesterolemic plus *E.faecium*; HI = hypercholesterolemic plus isoflavone.

Animals that received soy yogurt exhibited lower concentrations of oxLDL Ab (P < 0.05) than group H, after 30 days of treatment. At the end of the experiment, oxLDL Ab was significantly higher (P < 0.05) in group H (Table 3). The oxLDL Ab level was also compared with the baseline value (T0) for each animal group (Figure 1). The rabbits fed a high cholesterol diet (H), placebo (HP) and isoflavone mixture (HI) exhibited a rapid rise in oxLDL Ab (P < 0.05), while soy yogurt and soy yogurt supplemented with isoflavones reduced the percent increase of this parameter during the course of the experiment. Interestingly, the *E. faecium *suspension increased the oxLDL Ab in the first 30 days (P < 0.05), but during the later period of treatment the concentration fell to a level near those of groups C, IY, HIY and HY (Figure 1).

**Table 3:** Variation in levels of autoantibodies against oxLDL in plasma of rabbits.

Groups*	**Oxidized LDL autoantibodies (mg/L)**		
	
	**T0**	**T30**	**T60**
**C**	0.204 ± 0.104^a^	0.228 ± 0.140^b^	0.316 ± 0.156^b^
**IY**	0.272 ± 0.138^a^	0.407 ± 0.137^ab^	0.409 ± 0.137^b^
**H**	0.338 ± 0.166^a^	0.821 ± 0.284^a^	1.809 ± 0.514^a^
**HIY**	0.338 ± 0.166^a^	0.531 ± 0.267^ab^	0.530 ± 0.232^b^
**HY**	0.390 ± 0.289^a^	0.391 ± 0.097^b^	0.553 ± 0.296^b^
**HP**	0.323 ± 0.105^a^	0.671 ± 0.223^ab^	0.799 ± 0.285^b^
**HE**	0.171 ± 0.050^a^	0.550 ± 0.243^ab^	0.354 ± 0.177^b^
**HI**	0.220 ± 0.089^a^	0.527 ± 0.085^ab^	0.556 ± 0.111^b^

Values (in mg/dL) represent mean ± SEM (n = 6). Statistical comparison of groups: means with identical letters in the same column do not differ significantly (P ≤ 0.05). *C = control; IY = isoflavone supplemented soy yogurt; H = hypercholesterolemic; HIY = hypercholesterolemic plus isoflavone-supplemented soy yogurt; HY = hypercholesterolemic plus soy yogurt; HP = hypercholesterolemic plus placebo; HE = hypercholesterolemic plus *E.faecium*; HI = hypercholesterolemic plus isoflavone.

### Sudanophilic lesions in rabbits aortas

**Figure 2:**
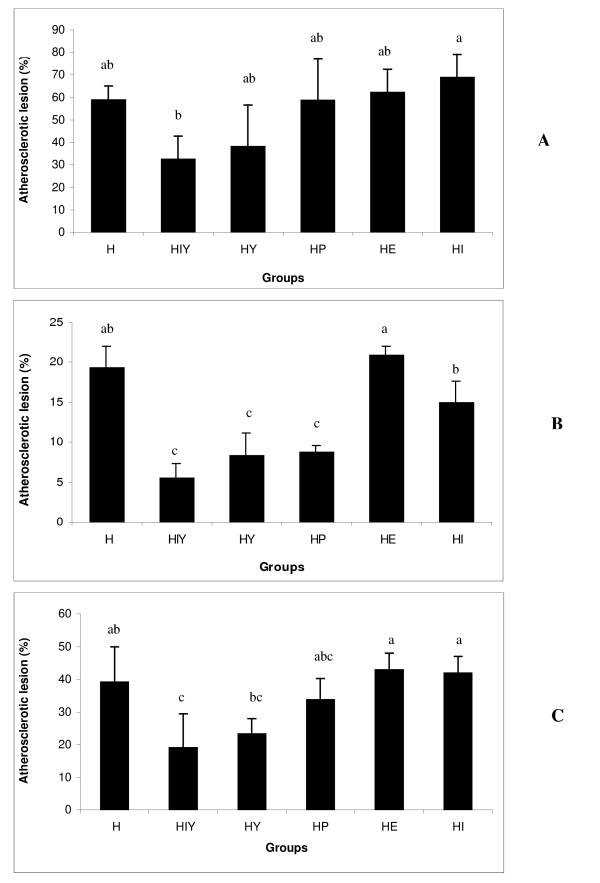
**Percentage of aortic area - A = arch segment; B = thoracic and abdominal segments; C = whole aorta - covered with lesion**. The bar graphs represent the average (n = 6) for each group with standard errors. H = hypercholesterolemic; HIY = hypercholesterolemic plus isoflavone-supplemented soy yogurt; HY = hypercholesterolemic plus soy yogurt; HP = hypercholesterolemic plus placebo; HE = hypercholesterolemic plus *E.faecium*; HI = hypercholesterolemic plus isoflavone.

Comparisons of cross-sectional atherosclerotic lesion area in aortic segments are shown in Figure 2. All rabbits on the cholesterol-enriched diet developed atherosclerosis. In contrast, no animals fed a normal diet developed atherosclerotic lesions. No significant difference was observed in the lesion area in the aortic arch (%), relative to group H. The extent of atherosclerosis in the thoracic and abdominal aorta was reduced in groups HIY, HY and HP. However, when the whole aorta was analyzed, animals treated with soy yogurt supplemented with isoflavones exhibited the greatest reduction (51.4% compared to group H, P < 0.05) in atherosclerotic lesion area, without differing from HY and HP groups (40.8% and 13.9%, respectively, compared to group H, P > 0.05).

## Discussion

Dietary changes are recommended as the first line of intervention for moderate dyslipidemia. This study evaluated the potential cardiovascular protective effects of an isoflavone-supplemented soy yogurt. We also investigated the isolated contributions of the probiotic microrganism and isoflavones to the observed effects.

Feeding excessive amounts of cholesterol to rabbits induces rapid development of hyperlipidemia and atherosclerosis [9,27,28]. In this study, the cholesterol added to the diet induced hypercholesterolemia in all experimental groups and none of the treatments applied to these groups reduced the serum lipids to basal levels.

In this study, we observed a decrease in the total (26.6%) and non-HDL cholesterol (27.8%) in rabbits fed the placebo product (unfermented soy product), suggesting that the ingredients of the soy yogurt mixture may contribute to this effect. The ingredients used to obtain the placebo and the soy yogurt included, in each 100 g, 3.39 g of soy protein and 5.24 mg of total isoflavones, components that have potential cholesterol-reducing effects [29].

Though no significant differences were observed in TC or n-HDL-C levels of animals treated with placebo, soy yogurt and isoflavone-supplemented soy yogurt, the product fermented with *E. faecium *CRL 183 promoted the greatest reduction (P > 0.05) in TC and non-HDL-C (38.1% and 38.7%, respectively). The supplementation of soy yogurt with isoflavones did not enhance its hypolipidemic effect. In an earlier investigation of our group, using rabbits with diet-induced hypercholesterolemia, the serum TC content was reduced about 18% by ingestion of the unsupplemented soy yogurt [18]. In another study, rats fed fermented soy product enriched with isoflavones had significantly lower (P < 0.05) serum total cholesterol (15.5%) than rats fed on a hypercholesterolemic diet. Non-HDL cholesterol was lower (P < 0.05) in the rats fed fermented soy product, with (27.4%) or without (23.2%) added isoflavones, than in untreated hypercholesterolemic rats [30].

In a previous study, *E. faecium *CRL 183 reduced the cholesterol by 54% in an *in vitro *model [16]. However, the results of the present study did not confirm this effect in rabbits. The *E. faecium *culture did not promote any improvement in TC or n-HDL-C, indicating that the probiotic microrganism alone was not responsible for the beneficial effects of soy yogurt on these lipid fractions.

Epidemiological studies have demonstrated that HDL-C is a strong, independent, inverse predictor of cardiovascular disease risk [31-33]. The soy yogurt, supplemented or not with isoflavones, placebo and the *E. faecium *culture prevented the reduction of HDL-C levels, relative to the control group, and raised the HDL-C concentration relative to group H. The probiotic microorganism (*E. faecium *CRL 183) seems to be decisive for the improvement of the HDL-C level, since in the placebo group the reduction of this fraction in the blood was higher. In a previous experiment with rabbits, the HDL-C level increased 17.8% in the group that consumed the fermented product [18]. Also, the same fermented product, without isoflavone supplementation, was capable of raising the HDL-C level by 10% in normocholesterolemic men [19]. The increase of HDL-C could be an important health benefit of soy yogurt, since each 1-mg increase in HDL-C is associated with an estimated 2-3% reduction in cardiovascular risk [34].

The triglycerides level varied (P < 0.05) between groups at the beginning of the study, probably because of high biological variability of this lipid. In the current study, rabbits fed the pure culture of *E. faecium*, soy yogurt supplemented with isoflavones or placebo showed triglycerides concentrations significant lower, after 60 days on these diets. In a previous study with the same fermented product, with or without isoflavones, no effect on triglycerides levels was detected [18,19,30].

The differences in the magnitude of the observed effects on blood lipid levels, between the present and earlier studies that evaluated the same soy yogurt, might be explained by differences in experimental design. An important difference between the previous and present studies is the cholesterol concentration added to the diet to induce hypercholesterolemia and atherosclerosis. Rossi et al. [18] induced hypercholesterolemia by feeding the rabbits a cholesterol-enriched diet (0.15% wt/wt) during the first 15 days of the experiment. In the present study, rabbits were fed a cholesterol-enriched diet for the entire 60 days (1% wt/wt during the first 30 days and 0.7% wt/wt thereafter) to induce atherosclerotic lesions. The TC concentration of group H after this time was about 11 - fold higher than that observed in the earlier study and this could influence the effect of the soy yogurt on lipid parameters.

There are numerous reports showing the hypocholesterolemic effects of probiotics. Xiao et al [12] showed in rats that milk fermented with *Bifidobacterium longum *BL1 reduced the total and LDL cholesterol, while no change in HDL-C concentration was observed. Paik et al [11] found that *Bacillus polyfermenticus *SCD reduced plasma LDL-C and triglycerides in rats after 6 weeks of treatment. The mechanisms involved in the reduction of serum cholesterol by probiotics, although not completely known, include deconjugation of bile salts, cholesterol assimilation by probiotic cells and fermentation of indigestible carbohydrates from the diet to produce short-chain fatty acids [35,36]. The lipid profile effects observed here probably involve the ingredients of soy yogurt, the probiotic microorganism and its bioactive metabolites.

The isoflavone mixture reduced the TC and n-HDL-C (19.5%) after 30 days of feeding. However, this effect was not maintained until the end of the protocol. Several studies have assessed isoflavones as a hypocholesterolemic agent and the results are contradictory. Antony et al [37,38] showed that soy protein without isoflavones was less effective at lowering blood cholesterol levels and preventing the development of atherosclerosis in monkeys than isoflavone-rich soy protein. In contrast, Adams et al [39] showed that monkeys fed a soy protein diet with low isoflavone content exhibited a lipid profile similar to those fed a similar diet high in isoflavones. Wilson et al [40] observed that soy protein, with or without isoflavones, lowered TC and n-HDL-C concentrations in hamsters.

The association between oxidized LDL, hypercholesterolemia and atherosclerosis has been demonstrated in numerous studies in animals and humans [15,41]. Oxidative modification of LDL induces the formation of immunogenic epitopes in the LDL, leading to the generation of antibodies against oxidized LDL (oxLDL Ab) [42]. In this study, the formation of autoantibodies against ox LDL was higher (P < 0.05) in the hypercholesterolemic group (H) by the end of the treatment and the intake of soy yogurt, supplemented with isoflavones or not, prevented this rise in oxLDL Ab during the experiment. The reduction in non-HDL-C concentrations (LDL+VLDL+IDL), with a consequent reduction in LDL particles available for oxidation, observed in the HY and HIY groups could, partially, explain this effect.

The importance of autoantibodies against oxLDL in atherogenesis remains controversial because of the complexity of oxLDL Ab system [4]. An atherogenic role was supported by studies that found elevated concentrations of oxLDL Ab in patients with atherosclerosis [43-45]. Conversely, other studies found an inverse relation between autoantibodies and atherosclerosis development [4,46]. Analyzing the whole aorta of the rabbits, we observed that only soy yogurt supplemented with isoflavones reduced the atherosclerosis. The variables analyzed in this study are insufficient to define the exact causes or mechanisms involved in the antiatherogenic effect of the soy yogurt. However, given that atherosclerosis is a chronic immune inflammatory disease, it is possible that increase in levels of antibodies against oxidized LDL in the H, HP, HE and HI groups is linked to the development of the atherosclerotic lesion. On the other hand, the results indicate that the soy yogurt supplemented with isoflavones had an atheroprotective effect that may include the anti inflammatory properties of the probiotic product already described [47] and the antioxidant effect of isoflavones [14].

## Conclusion

In conclusion, this study showed that the soy yogurt could be used as an alternative means of reducing the risk of cardiovascular diseases by increasing HDL-C, lowering total cholesterol and n-HDL-C and inhibiting oxLDL Ab formation. Our findings also suggest that isoflavone supplementation may enhance the antiatherosclerotic effect of the soy yogurt.

## Abreviations

CVD: Cardiovascular Disease; (C): control group; (H): hypercholesterolemic group; (IY): isoflavone-supplemented soy yogurt group; (HY): hypercholesterolemic plus soy yogurt group; (HIY): hypercholesterolemic plus isoflavone-supplemented soy yogurt group; (HP): hypercholesterolemic plus placebo group; (HI): hypercholesterolemic plus isoflavone group; (HE): hypercholesterolemic plus *E.faecium *CRL 183 group; TC: Total Cholesterol; HDL-C: High Density Lipoprotein Cholesterol; LDL-C: Low Density Lipoprotein Cholesterol; VLDL: Very Low Density Lipoprotein Cholesterol; n-DHL-C: Non High Density Lipoprotein Cholesterol; oxLDL: oxidized Low Density Lipoprotein; oxLDL Ab: Autoantibodies against oxidized Low Density Lipoprotein.

## Competing interests

The authors declare that they have no competing interests.

## Authors' contributions

DCUC was involved in design, data collection, drafting the manuscript and revising it critically for important intellectual content. DSPA, RCV, RB, LQB, NDPS, GFV were involved in data collection and drafting the manuscript. EAR was involved in design, drafting the manuscript and revising it critically for important intellectual content.
